# The Use of Alcohol in Medical Practice

**Published:** 1874-07

**Authors:** John Morris

**Affiliations:** Baltimore.


### ARTICLE III.

### *The Use of Alcohol in Medical Practice.*—Concluded.

BY JOHN MORRIS, M. D., OF BALTIMORE.

A report read before the Medical and Chirurgical Faculty of Maryland.

Surgeon General Maclean, of the British army, in a recent article on this subject is most sweeping in his condemnation of the use of alcohol in the army.

He says: "If there be any point of military hygiene that now may be regarded as settled beyond doubt or cavil it is this, that spirits are not only not helpful, but are hurtful to the marching soldier everywhere, but nowhere more so than in hot climates. The evidence shows that wherever soldiers, by accident or design, have been cut off from the use of spirits on marches, in active service, in temperate climates exposed to wet and cold, or in tropics to ardent heat, or in laborious sieges, they have maintained their health, spirits, discipline, far better than when the once deemed indispensible grog was in daily use."

His colleague in the military school, Doctor Parkes, and the late Count Wallowicz, in a series of careful experiments on the use of alcohol, carried on at Netley, have placed on a sure scientific basis what was before a matter of observation, and have established that alcohol, far from increasing the power of bearing fatigue, even when given in a quantity which many spirit drinkers would deem within the limits of moderation, lessens muscular force, and a quantity in excess of this, it was shown, entirely destroyed the power of work.

Doctor Maclean further says: "I cannot leave this important subject without adding that for twelve years I have at Netley had unrivalled opportunities of studying the effects of habitual dram-drinking on the persons of our soldiers, and I add my testimony to the immense weight of evidence accumulated by medical men in civil and military life, to the effect that alcohol is one of the most active agents in causing the degeneration of the human tissues—in other words disease, premature decay and death."

The conclusions of Dr. Parkes and Count Wallowicz, before alluded to are thus stated. The effects on appetite and on circulation are the practical points to seize, and if we are correct in our inferences, the commencement of narcotism marks the point when both appetite and circulation begin to be damaged. As to the metamorphosis of nitrogenous tissues, or to animal heat, it seems improbable that alcohol in quantities that can be properly used in diet has any effect. It appears to us unlikely (in the face of the chemical results) that it can enable the body to form more work on less food, though, by quickening a failing heart, it may enable work to be done which otherwise could not be done. It may then act like a spur in the side of the horse, eliciting force, though not supplying it.

Even Dr. Dupre, a most earnest supporter of Dr. Austie's views, only *assumes* that it is food. He says: "On consideration it will be found that the observed diminution of urea following the consumption of alcohol is strong evidence in favor of the assumption that alcohol is a food, and that to a certain extent, as far as the production of force, for example, is concerned, it is really capable of taking the place of food, and therefore protecting the tissues from the wear and tear of life."

The advantages of abstinence from alcohol during exposure to cold is a most striking fact. A group of men, twenty-six in number, travelling over the Plains, lost their way and were overtaken by darkness. The weather was intensely cold, and only three of the twenty-six abstained from alcohol. Dr. McKinley, who accompanied the expedition, tells the fate of the party in a few words, in a communication to a Cincinnati medical journal: Those that got drunk, froze dead, those that drank less, but too much, died after a while; those who drank only moderately, will feel it as long as they live; out of the twenty-six only the three survived who abstained from alcohol. All were equally well provided, each having two blankets. All were in the bloom of life, and the best of health, and ready to encounter and able to overcome the hardships inseparable from a frontier life. This only confirms the views of all travellers, includ ing such Arctic explorers as Ross, Richardson, Franklin, Kane, Hayes, etc. Indeed, the Hudson Bay Company prohibit the use of alcohol by all persons in its employ. From these facts it may clearly be inferred that alcohol is not only not a food, but a poison—a poison as much as opium, and should be administered with the same caution, and sold in the same manner and under the same legal restraints.

Dr. Forbes Winslow, whose valuable life has just been lost to the profession, in his testimony before the parliamentary committee, says: "Alcohol is not a necessary of life. It should be dealt with by the legislature as a poison. A person goes into a dram shop and takes his rum or whiskey; he imbibes a poison. After a time his nervous system becomes saturated with it, and the brain itself becomes surcharged with alcohol, and, as is the case very often with chronic drunkards, on examination after death, if you apply a light to the venticles of the brain, it ignites into a flame. You can actually distil alcohol from the brain of chronic drunkards; the brain is so saturated with the spirit, and, of course, the whole source of vitality becomes poisoned."

The two next questions: Is alcohol necessary in the treatment of disease, and is the good it works at all equal to the injury it produces? are now to be considered. For our own part, we do not deem alcohol absolutely necessary as a means of cure. It can be supplemented by other agents, simple and inocuous. The ethers can be employed in many cases in which alcohol is now administered, and with equal efficacy. Their use involves no liability of the habit of taking them being created, for as much as they are nauseous and unpleasant, and could scarcely come into common use as a stimulant. Milk is a far better remedy in typhoid fever, and, in connection with raw beef, is much superior in the treatment of all wasting diseases. Digitalis, bromide of potassium, quinine, more than fill its place in heart troubles, and affections of the nervous system. For fatigue and nerve enervation, coffee, as we before stated, is above all our best and most reliable agent. It is admitted even by those who contend for the use of alcohol in fevers, that there are subjects to whom it is injurious—patients in whom the secondary digestive process within the blood is slowly performed. The nervous depression induced by this condition, it is conceded, more than counterbalances any nutritive advantage it may possibly possess. It may then fairly be presumed that there are few cases of disease in which alcohol cannot be supplanted by some other remedy within our reach. The good that it may possibly effect in a few forms of disease, cannot be used as a set-off to the incalculable amount of physical degeneration produced by its use, independent of the moral consequences, which it is not the purpose of this paper to discuss.

Now let us consider how far medical men are responsible for its use as a dietetic agent. We must not be understood to assert that any great part of the intemperance of the day is due to the medical profession; indeed, we feel convinced that the cases are very few in which drunkenness has occurred from the inconsiderate prescription of alcohol. Above all have we no patience with those disingenous and cowardly persons, particularly clergymen, who attribute their intemperate habits to the prescription of stimulants. It is due to the truth, however, to state, that so great an authority as the *Lancet* admits that in some instances this is the case in England, particularly among the higher classes of women.

A great deal of harm has been done we are convinced by the mere fact of calling alcohol a stimulant. It is not a stimulant in the true sense of the term. as we have already shown. The popular mind, however, has been taught to believe that it is, and men and women rush to it in the firm persuasion that its powers are beneficent and life-sustaining, who would avoid its use did they know that it depresses all the vital forces, and tends to general and permanent enfeeblement; and that it only has the power to evoke the will to performance without conferring the necessary strength. This fact is demonstrated in the modern treatment of delirium tremens. Under the old system the gradual method was adopted, and the patient and his friends were induced to believe that the sudden withdrawal of alcohol would inevitably lead to fatal consequences, that is: That a patient already feeble and prostrate was to be still further subjected to the influence of the agent, which had been the cause of all his suffering. By the modern plan, in which alcohol is totally prohibited, a patient recovers in one-half or even one-third of the time, without the progress of his case being marked by those painful and melancholy symptoms which attended the old method. Food administered frequently and in small quantities, is, in fact, the only proper remedy for chronic alcoholism; it arrests degeneration of nerve tissue, and also relieves the intense sinking and craving for stimulants so generally experienced by drinkers.

Debility, as we before stated, and anœmia are ever before the eyes of certain practitioners, and serve as an excuse for alcoholic medication, and these terms, we are sorry to say, are frequently used to hide a great deal of ignorance.

The *Lancet* is very severe on this class of medical men. It declares its belief that all the mischief in the administration of alcohol in chronic disease is due to the unthinking and illogical prescription of it for debility, merely as such. It adds: "It is no figure of speech, but the literal truth, when we say that hundreds of neuralgic, hysteric and epileptic patients have been driven into drunkenness or lunacy, or both, by the careless folly of advisers, who had no better reason for the prescription of alcohol than the fact that these diseases are attended with nervous weakness, as they undoubtedly are. The assumption involved—that so much ingested alcohol is necessarily so much added nervous strength—is so gross a fallacy that no one would assent to it if expressed in plain words. Yet we constantly see it acted upon. We repeat, with all the energy of which we are capable, that it is a grave scandal and mischief that medical men should endanger in this serious way the powers of moral resistance, of women and other weak persons, while basing their practice upon ideas that are illogical and untenable, and we trust that a reform in this respect will be immediately commenced.

Be this as it may, we believe medical men have it in their power to do much to restrain the use of alcohol. The public believe that it is a necessary article of diet: that it is highly nutritious and strengthening, and that if actual drunkenness does not supervene from its use it cannot be taken too freely—this is the great error which it is our office and duty to correct. Doctor Austie's investigations fix one and a half ounces of absolute alcohol, that is, three ounces of proof spirits as the quantity that an adult can take daily without actual injury. Dr. Parke's experiments proved that two ounces affected a strong, healthy soldier, with what may be fairly called symptoms of poisoning.

The safe rule in using alcoholic liquors, then, may be laid down as a safe rule for the guidance of the people, that one and a half pint of ale, containing five per cent. of alcohol, or a pint of claret or other light wine, containing ten per cent., is the exact amount that may be taken for dietetic use without detriment. Let the public, then, be taught, through us, that every drop taken beyond this is poisonous; that every drop taken beyond this is destructive of life force; that every drop taken in excess of this tends to enervation of mind and body, and if continuously taken, will lead to premature decay and death. This kind of teaching the community requires. It may not make men tee-totalers, but it will tend to make them temperate. There is no disagreement in our profession concerning the action of alcohol on the heart, stomach, nerves or other organs, and it is on these points particularly that the people most need enlightenment. It is not necessary for medical men to ally themselves with any party on this subject either in religion or politics, because by doing so they may lessen their influence as public teachers; but it is their solemn duty to enforce habits of temperance, and to lay down such rules for the guidance of their patients in the matter of diet as will secure their health and happiness. The whole subject rightly belongs to them, and it is only to them that proper reforms can be instituted or enforced, and the sooner this is discovered, both in Church and State, the better will it be for mankind, and the sooner may we hope to see a true advance made in the civilization of the age.

The paper by Dr. Morris was warmly applauded.

